# Whole-genome resource sequences of 57 indigenous Ethiopian goats

**DOI:** 10.1038/s41597-024-02973-2

**Published:** 2024-01-29

**Authors:** Shumuye Belay, Gurja Belay, Helen Nigussie, Han Jian-Lin, Abdulfatai Tijjani, Abulgasim M. Ahbara, Getinet M. Tarekegn, Helina S. Woldekiros, Siobhan Mor, Keith Dobney, Ophelie Lebrasseur, Olivier Hanotte, Joram M. Mwacharo

**Affiliations:** 1 Tigray Agricultural Research Institute, Mekelle, Tigray Ethiopia; 2 Addis Ababa University, Department of Microbial, Cellular and Molecular Biology, Addis Ababa, Ethiopia; 3 LiveGene Program, International Livestock Research Institute (ILRI), Addis Ababa, Ethiopia; 4 ILRI-CAAS Joint Laboratory on Livestock and Forage Genetic Resources, Beijing, China; 5 Animal and Veterinary Sciences, Scotland’s Rural College (SRUC), Roslin Institute Building, Midlothian, UK; 6 Department of Zoology, Misurata University, Misurata, Libya; 7 Institute of Biotechnology, Addis Ababa University, Addis Ababa, Ethiopia; 8 Department of Anthropology, Washington University in St. Louis, St. Louis, Missouri USA; 9 Institute of Infection, Veterinary and Ecological Sciences, University of Liverpool, Liverpool, UK; 10 Department of Archaeology, Classics and Egyptology, University of Liverpool, Liverpool, UK; 11 School of Philosophical and Historical Inquiry, University of Sydney, Sydney, Australia; 12 School of Life Sciences, University of Nottingham, Nottingham, UK; 13 Small Ruminant Genomics, International Centre for Agricultural Research in the Dry Areas (ICARDA), Addis Ababa, Ethiopia

**Keywords:** Animal breeding, DNA sequencing, DNA sequencing

## Abstract

Domestic goats are distributed worldwide, with approximately 35% of the one billion world goat population occurring in Africa. Ethiopia has 52.5 million goats, ~99.9% of which are considered indigenous landraces deriving from animals introduced to the Horn of Africa in the distant past by nomadic herders. They have continued to be managed by smallholder farmers and semi-mobile pastoralists throughout the region. We report here 57 goat genomes from 12 Ethiopian goat populations sampled from different agro-climates. The data were generated through sequencing DNA samples on the Illumina NovaSeq 6000 platform at a mean depth of 9.71x and 150 bp pair-end reads. In total, ~2 terabytes of raw data were generated, and 99.8% of the clean reads mapped successfully against the goat reference genome assembly at a coverage of 99.6%. About 24.76 million SNPs were generated. These SNPs can be used to study the population structure and genome dynamics of goats at the country, regional, and global levels to shed light on the species’ evolutionary trajectory.

## Background & Summary

Archaeological evidence indicates that all domestic goats (*Capra hircus*) derive from the wild bezoar *(Capra aegagrus*) that was domesticated in the central Iranian Zagros Mountains and/or Southeastern Anatolia about 10,000 years ago, making them the first livestock animal to be herded by early farmers^1,2^. The world has a population of more than one billion domestic goats^3^ and some 576 breeds^4^. Asia and Africa are ranked first and second with 59.4% and 35.0%, of the world’s goat population, respectively^5^, whilst Ethiopia is ranked second in Africa after Nigeria (https://www.statista.com/statistics/1290087/goat-population-in-africa-by-country/). An estimated 52.5 million goats are found in Ethiopia, and nearly all (99.9%) are indigenous genotypes reared by smallholder sedentary agro-pastoral farmers and pastoralists^6^. These indigenous goats are known for their adaptive resilience to diverse environments and production systems^7,8^. Because of their ease of management, and minimal initial capital investment, indigenous goats are preferred by smallholder farmers and pastoralists in contrast to cattle. In addition, their socio-economic, nutritional, and cultural significance means that indigenous goats are essential household assets to most African communities.

Although indigenous goats are a significant genetic resource to most agricultural households in Africa and the majority of developing countries, their genetic improvement has been hindered by their lack of systematic characterisation at the phenotypic and genetic levels.

Africa is home to a large genomic reservoir of indigenous goat populations of diverse phenotypes (see Breeds | DAGRIS (cgiar.org). While previous research has been undertaken on the genetics of African indigenous goats using microsatellite^9–18^ and SNP microarray genotypes^19–23^, relatively few studies have been conducted on these breeds using whole-genome sequencing (WGS) information. For example, for the African continent, WGS are only publicly available in the vargoats database (https://www.goatgenome.org/vargoats.html) including for Ethiopia (73 genome sequences of eight breeds)^24^, Morocco (44 genome sequences from three breeds)^25^, Kenya (15 sequences from two breeds), Madagascar (35 sequences from four breeds), Mali (36 sequences from six breeds), Malawi (24 sequences from five breeds), Mozambique (23 sequences from five breeds), Tanzania (39 sequences from five breeds), Uganda (three sequences from one breed), Zimbabwe (20 sequences from two breeds) and Nigeria (three sequences from two breeds) (https://ncbi.nlm.nih.gov/). These publicly accessible genome data are important for (i) studying population-level genetic diversity and structure, (ii) understanding domestication and evolutionary history, (iii) detecting adaptation selective sweeps, and (iv) discovering variants (SNPs, structural variants, causative mutations e.t.c.) to address goat breeding challenges and boost goat farming in the continent.

Our study presents new WGS data of 57 indigenous Ethiopian goats from 12 populations, comprising ~2 Tb of raw sequence data. It is by far the most representative dataset of whole genome sequences for goats found in any African country considering a high number of breeds from highly diverse agro-ecosystems. This data includes ~24.76 million usable SNPs that passed rigorous quality control filters, of which approximately 30% are novel. This is a valuable addition of genomic resources to the caprine biological repository in the continent and the globe. It provides an opportunity to detect potential novel SNPs compared to the 50 K SNP chip array previously reported in African goat populations^19–23^. It also provides a new avenue that facilitates better understanding of salient genomic features (e.g., genes, coding sequence, regulatory regions, pseudogenes, repeat sequences) and/or uncover candidate genomic regions controlling traits of production, reproduction, and adaptive significance. Moreover, the resource can be used to identify (albeit tentatively) opportunities and threats of genetic diversity, which can be used as baseline information to design strategic options for future sustainable utilization of the species. However, ensuring high-quality data with representative samples and performing accurate quality control procedures is of critical importance before one can proceed with mapping against reference genome assemblies, and making the data accessible to the public and opening the door to further research. In this article we present the entire process we used to achieve accurate quality control measurements and procedures from raw data to the final variant call format (VCF) file generation while minimising false positives and detecting true variants.

## Methods

### Sample collection, dna extraction and quality control

**Fig. 1:**
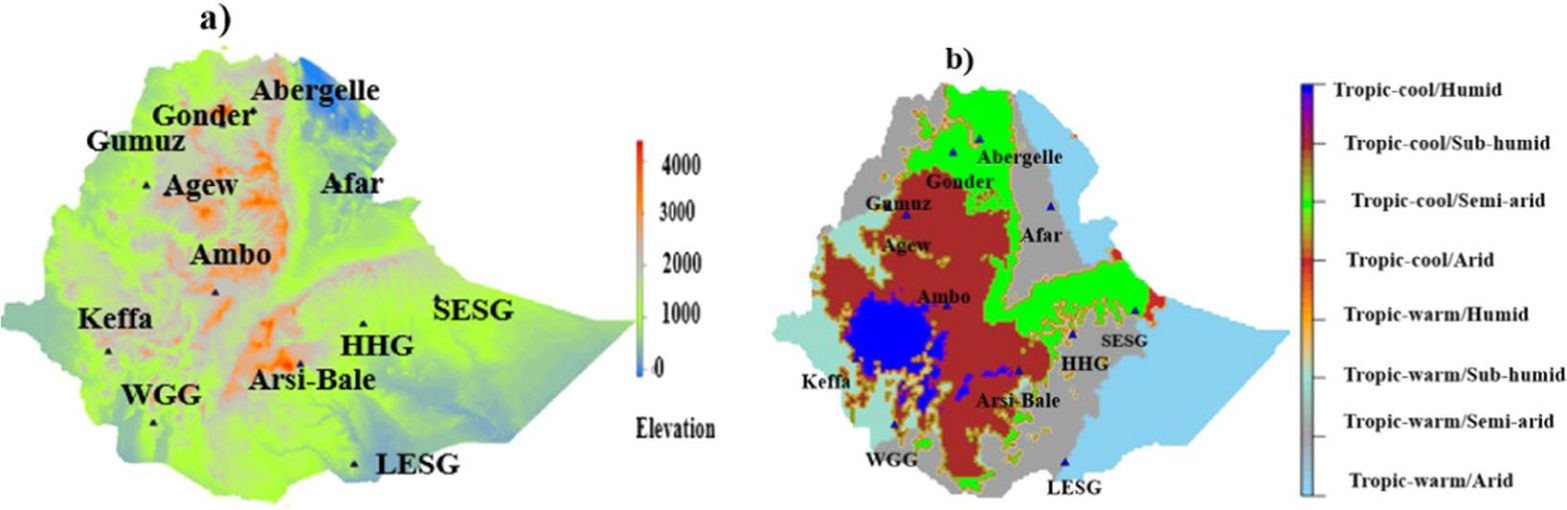
Map of the study areas representing the geographic distributions of indigenous Ethiopian goat populations based on: (**a**) Elevation, and (**b**) Agro-ecological zones and climatic conditions. Abbreviations: HHG= Hararghe Highland Goats, LESG=Long Ear Somali goats, SESG=Short Ear Somali goats, and WGG= Woyto-Guji goats.

Genomic DNA was extracted from the whole blood of 57 genetically not unrelated individuals (only one individual was sampled per flock)^23,26^, of 12 indigenous Ethiopian goat populations from diverse agro-eco-climatic zones (Fig. 1, Table 1). The working hypothesis was that these 12 indigenous goat populations are adapted to their production environments’, agroecological and climatic conditions and thus represent distinct genetic units. The genomic DNA was whole-genome sequenced at a depth of ~10x and read length of 150 bp paired-end following library construction, on the Illumina 1.9 NovaSeq 6000 platform (https://en,novogene.com/services/reserachservices/genome-sequencing/whole-genome-sequencing-wgs/). The initial base call files were converted into FASTQ files in the sequencing library prior to quality pruning using the bcl2fastq software^27^. The sequencing company performed the first stage QC of the FASTQ files using their in-house software. Secondary QC of the generated fasta.gz files was performed using the FASTQC package (v0.11.5)^28^. The output files (fastqc.zip) were then aggregated in one directory and a single report was generated and used to visualize and screen biases, and assess the overall sequence quality using the MultiQC package (v1.8)^29^.

**Table 1:** Samples of the Ethiopian indigenous goat populations based on their geographic and climatic distributions.

S. No	Population	No	Region (s)	District (s)	Altitude (m)	Latitude	Longitude	Rainfall (mm)	Tem. (°C)
1	Abergelle	4	TA	TAZ	900–1,800	13.20°	38.96°	370–700	21–41^48^
2	Afar	5	Afar	Melka-Werer	−125–2,870	11.94°	40.35°	92–673	27–41^49^
3	Arsi-Bale	5	Oromia	Arsi-Bekoji	2,780 (mean)	7.52°	39.25°	1,098 (mean)	7–19^50^
4	Ambo	5	Oromia	Meta-Robi	1,376–2,904	9.33°	38.19°	750–1,300	15–31^51^
5	Gonder	3	Amhara	Lay-Armachiho	1,550–1,800	12.40°	37.45°	711.8- 1822	13–28^52^
6	HHG	5	Oromia	Hirna	1,300–2,450	8.98°	41.27°	990–1,010	14–26^53^
7	Keffa	5	SWEPR	Tepi and Sheka	900–2,700	9.12°	32.42°	1,559 (mean)	15.5–29.7^54^
8	LESG	5	Somali	Filtu	200–1,500	5.25°	40.93°	400–600	25–40^55^
9	SESG	5	Somali	Kebri-Beyah	950–1,350	9.12°	43.18°	500–700	22.5–32.5^56^
10	Agew	5	Amhara	Addis-Kidame	2,400 (mean)	11.13°	36.86°	2,379 (mean)	11–25^57^
11	Gumuz	5	Benshangul	Pawe	1,500–2,500	11.33°	36.35°	500–1,800	27.50^58^(mean)
12	WGG	5	SNNPR	Konso	600–2,100	5.23°	37.43°	400–1,000	12–33^59^

SNNPR = Southern Nations, Nationalities, and Peoples Region, SWEPR = Southwest Ethiopia Peoples’ Region, TA = Tigray and Amhara, TAZ = Tanqua-Abergelle and Zuquala, SESG = Short eared Somali Goat, LESG = Long eared Somali Goat, Central highland goat (also called Ambo), HHG = Hararghe Highland Goats, WGG = Woyto-Guji Goats, Tem. = Temperature.

### Genomic alignment and variant calling

**Fig. 2:**
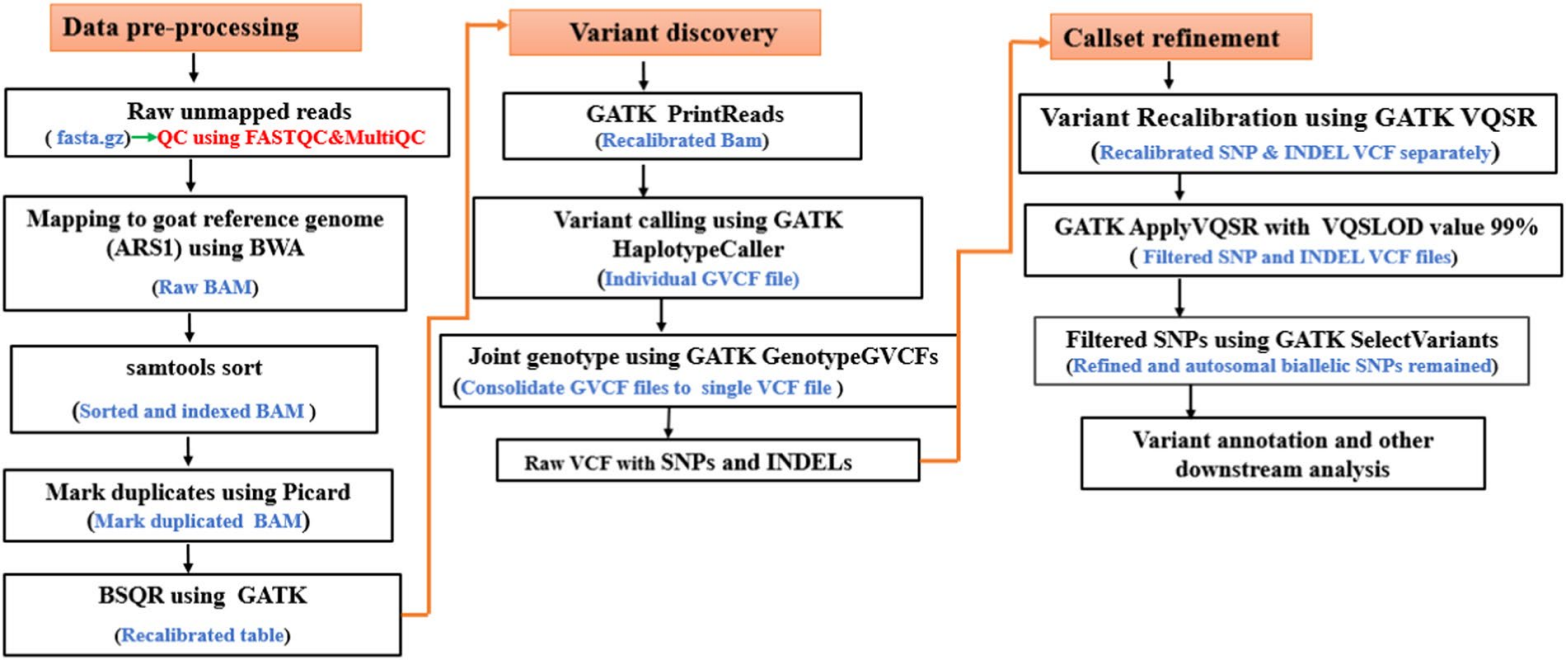
The overall workflow of the quality control procedure and parameters used across all the stages of DNA sequencing (data pre-processing, variant discovery, and callset refinement).

After ascertaining sequence quality, the paired-end reads were aligned to the goat reference genome assembly (ARS1; GenBank accession number GCA_001704415.1) using the Burrows-Wheeler Alignment tool (BWA-MEM v 0.7.17)^30^ for variant identification. The BAM files were sorted and indexed using SAMtools v1.8^31^. The function “MarkDuplicates” executed in Picard tool v2.18.2 (http://picard.sourceforge.net) was used to mark and discard flagged duplicates. After removing the duplicates, Base Quality Score Recalibration (BQSR), a data pre-processing step executed in GATK v3.8-1-0-gf15c1c3ef^32^, was used to estimate the accuracy of each base call and detect systematic errors arising from the sequencing process and generate recalibrated BAM files. The GVCF files for each sample were generated using the GATK HaplotypeCaller from the recalibrated BAM files. Finally, joint genotyping was performed and a single VCF file containing SNP and INDEL variants produced (Fig. 2).

### Variant filtration and genotype refinement

Variant Quality Score Recalibration (VQSR) step was performed using the knownSites of the ARS1.0 Ensembl version 99 (https://e99.ensembl.org/capra_hircus) and filtered out the bad and good variants using the GATK. Variant call annotations such as Read Depth, Quality of Depth, Fisher Strand Test, Mapping Quality Score, Mapping Quality Rank Sum Test, Read Position Rank Sum Test Statistic, StrandOddsRatio Test, mode SNP and the VQSRTranchesSNP90.00 to 100.00 were used. Using the ApplyRecalibration (ApplyVQSR) in GATK, a tranche sensitivity threshold of 99.0% was used to filter the variants. Finally, post-processing was conducted to remove variants failing the GATK filtering parameter thresholds and biallelic SNPs were extracted using ‘SelectVariants’ function with option “–selectType SNP-restrictAllelesTo BIALLELIC” as presented in Fig. 2. Here, only biallelic SNPs that passed filtration and can be used in downstream analysis are presented.

## Data Records

Whole-genome sequence data (FASTQ format) from 57 Ethiopian goat samples representing 12 populations analyzed herein have been deposited in NCBI under Sequence Read Archive (SRA) accession number SRP464279^33^.

## Technical Validation

### Quality control for raw reads

**Fig. 3:**
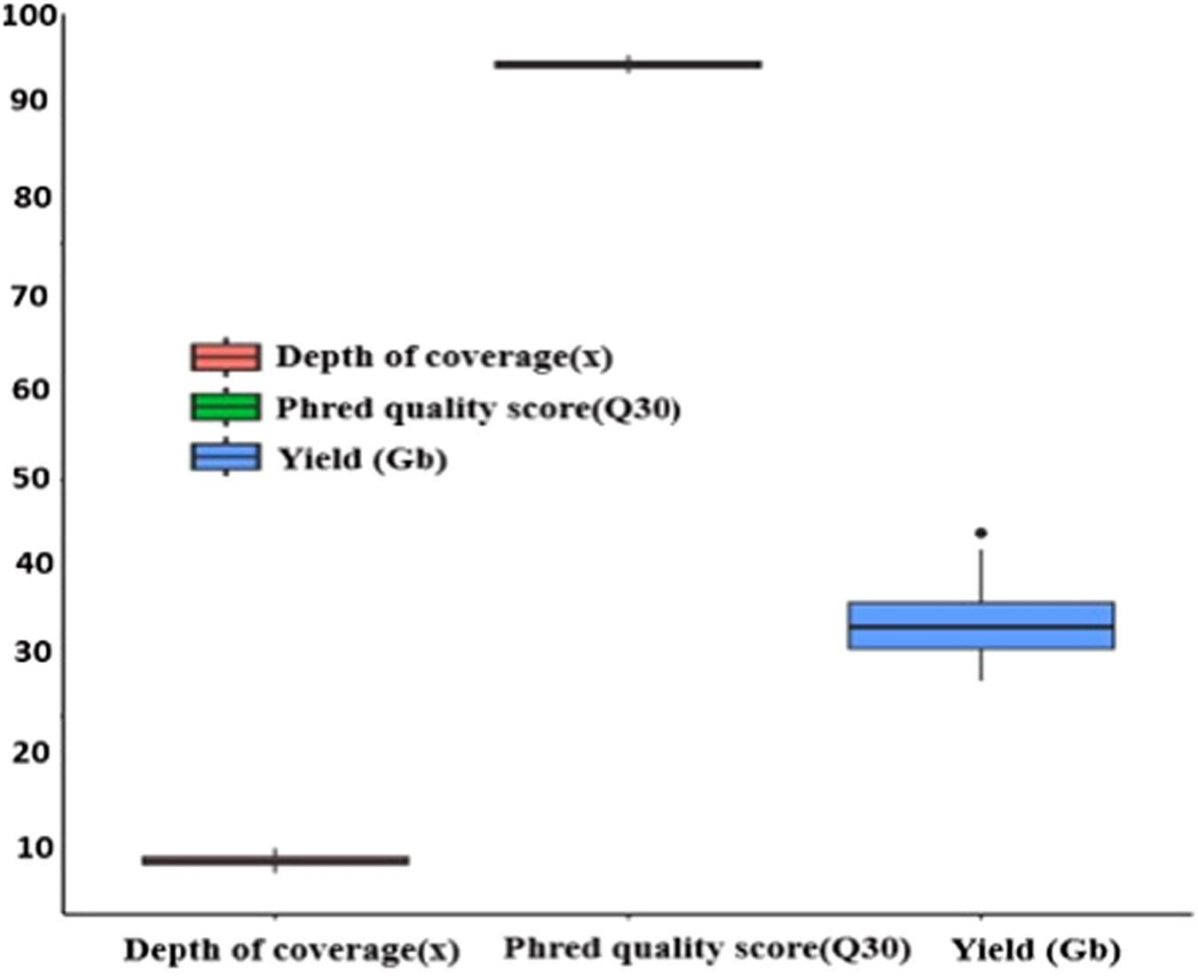
Boxplots showing the size of raw bases, Phred quality scores (Q30), and depth of coverage of the 57 indigenous Ethiopian goat genomes.

The Phred quality score is commonly used as a measure of the quality of the base-calls generated by automated DNA sequencing^34,35^. It is calculated with the formula^36^: Q = −10Log_10_^(E)^ where “Q” represents the base quality value, and “E” the error rate of the base recognition. The commonly used Phred-scaled base quality scores range between 2 and 40, with variations in the range depending on the origin of the sequence data^36^. A higher Phred score indicates a higher probability that a given base-call is correct, while the opposite is true. In our study, we used a Phred scaled score of 30 indicating the likelihood of an incorrect base-call once every 1000 bases equivalent to a precision rate of 99.9%. The raw bases of a sample ranged from 28.77 Gb to 44.43 Gb (mean ± SD = 34.97 ± 3.46 Gb), out of which 93–95% (mean ± SD = 94 ± 0.44%) of the samples had Phred scaled quality score of 30 (Fig. 3).

A depth coverage of greater than 4.4x has the power to identify novel variant calls. On the contrary, higher false-positive variants are amplified when the depth of coverage is lower than 4.4x^37,38^. In this study, the depth of coverage ranged from 8.38x to 11x (mean ± SD = 9.71x ± 0.60) (Fig. 3), which is an ideal depth for identifying variants accurately and achieved ~99.6% genome coverage and ~99.8% mapping success rate against the ARS1.0 goat reference genome assembly.

**Fig. 4:**
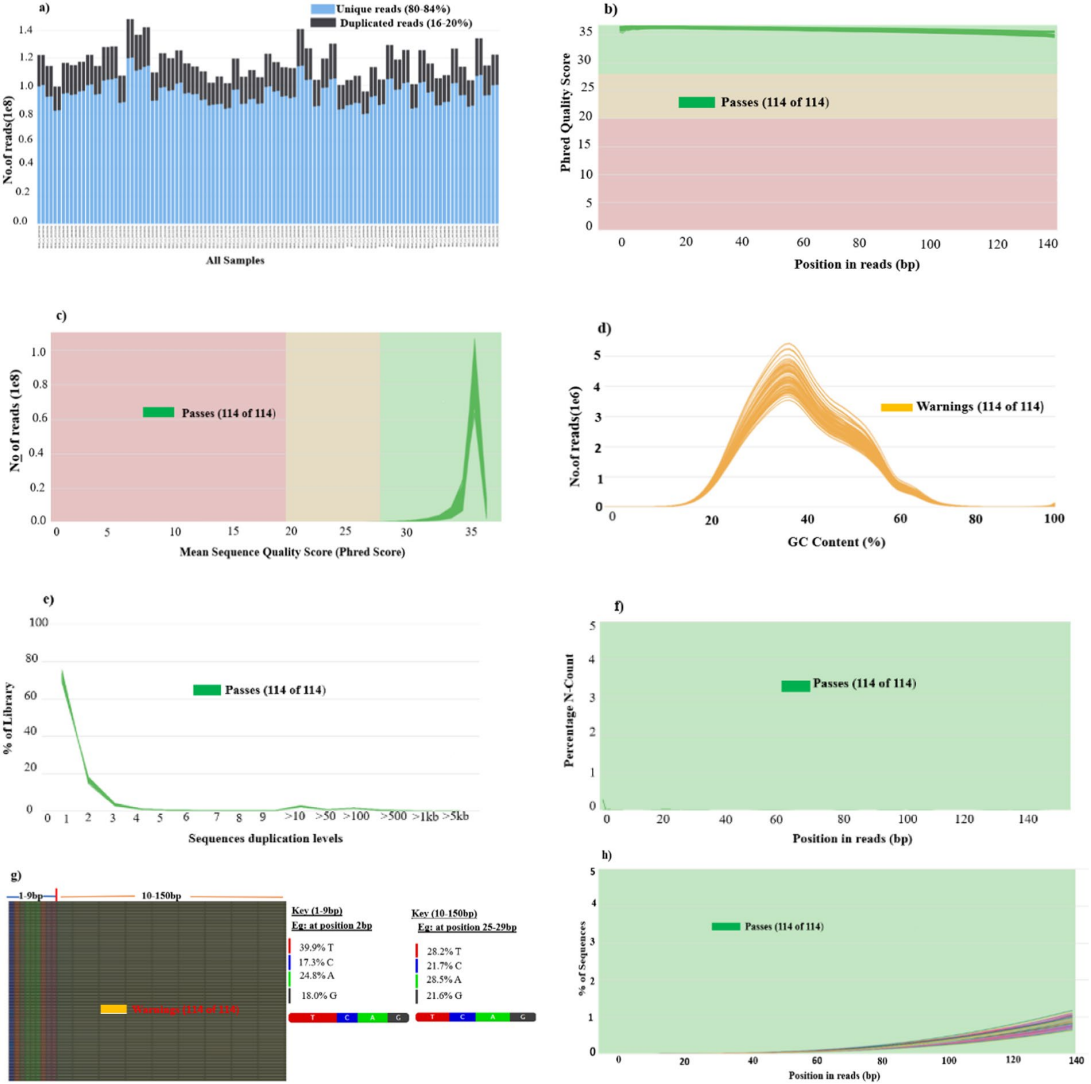
Quality control outputs of the high-throughput sequencing data of the 114 samples combined using the MultiQC package: (**a**) Unique and duplicated sequence counts, (**b**) Mean quality value across each base position in the read, (**c**) Per Sequence quality scores, (**d**) Per Sequence GC content, (**e**) Sequences duplication levels, (**f**) Per base N content, (**g**) Per Base sequence content (heatmap of the four nucleotide distributions: A, T, G, C), and (**h**) Adapter content.

Following the quality checks, we gathered the fasta.gz report for the 114 read samples (read 1 and 2) and run the MultiQC to generate a single report and identify good and bad samples. The report indicated that all the samples passed the QC parameters, such as base sequence quality score, sequence duplication level and per base N content, and confirmed the high-quality of our sequences (Fig. 4). For example, the level of duplication and unique sequence reads ranged from 16 to 20% and 80 to 84%, respectively (Fig. 4a). The low level of duplicated reads (<20%) indicate a high level of coverage of the target sequences. In contrast, higher values will show some kind of enrichment bias, such as arising from PCR artefacts, and/or biological duplicates^28^. However, all the QC parameters were assigned green signals, indicating high-quality sequencing standard. Out of the 114 reads generated, only 15 R2 reads showed warning signals (orange colour) of overrepresented sequences. However, these slightly abnormal reads have very low likelihood of affecting the quality of the SNPs and subsequent analysis. Generally, R2 reads have lower sequence quality compared to R1 reads^38,39^. This observation has been attributed to the fraction of the fragment length (>500 nucleotides) in the library independent of the tissue source, library type or sequencer model^39^.

The per base sequence content or heatmap of the distribution pattern of the four nucleotides (A, T, G, C) are flagged by a warning signal (Fig. 4g). In a random library, the normal expectation is that all four bases would be equally (25% of each base) and stably represented across all reads. This, however, is rarely the case as some genomes are either GC or AT rich. At the beginning of our sequences and taking the 2 bp position as an example, the difference between A and T, and G and C bases was 15.1% and 0.7%, respectively, indicating a biased distribution of the four nucleotides. If the difference between A and T, or G and C, in any position is greater than 10%, the per base sequence content will show a warning signal, while a fail signal will result if it is greater than 20%^28^. In Illumina platforms, the beginning and end of reads are more prone to low quality, which results in higher chances of false-positive calls^40^. However, from 10 to 150 bp and taking positions 25–29 bp in our sequences as an example, the difference between A and T, and G and C bases was 0.3% and 0.1%, respectively, which is lower than 10%.

Nevertheless, the overall heatmap depicting the distribution of the four nucleotides shows a slightly abnormal pattern but reasonable bases calls. This, however, has a low likelihood of affecting downstream analysis. This study observed no failed reads (no red signals) and unrecognized bases (N bases). The data can thus be used without QC procedures aimed at either removing adapters and/or poor-quality reads.

The per sequence GC content is another QC metric that is used to assess the quality of the length of each sequence^38^. Generally, the GC content differs across species and genomic region^40^. A normal random library typically has, more-or-less, a normal GC distribution content for all reads. An abnormal distribution could imply either a contaminated library or some systematic biase^28^. However, the GC plot of our data (Fig. 4d) is not a perfect normal distribution, and it is therefore not surprising that it is assigned a warning signal for all the 114 samples. This will however not affect the subsequent analysis. In this study, the mean GC content per sequence was 42.93%. If the GC content deviates from the average GC content by more than 5% and 10%, it results in a warning and failed signal, respectively^28^. The average GC content of the sequences generated herein approximates that reported in the animal kingdom (41.2%)^41^, and the goat reference genome assembly (42.7%)^42^ but is lower than the value reported for archaea (44.88%), bacteria (50.76%), and fungi (47.96%)^41^. Naturally, mycobacterial DNA is GC rich and more stable than that of mammalians.

### SNP Quality control

Following joint genotyping with GenotypeGVCFs, a total of 26.99 million markers were identified in the sex and autosomal chromosomes, including multiallelic SNPs. VQSR filtering was applied to remain with the actual variants. Further filtration was applied to the dataset using ApplyVQSR with a threshold value of 99.0%, indicating that we accept that 1.0% of the variants in the truth set may be incorrect. Following this filtration and the post-processing filtrations, 24.76 million autosomal biallelic SNPs were retained across the 57 samples. These were used to investigate population level genomic diversity, structure, and dynamics.

The total number of SNPs and annotated variants are presented in Supplementary Table 1. On average, 13.78 million SNPs, 1.65 million indels and 3.07 million novel variants were detected with no significant differences being observed between populations. These SNPs were annotated and an average of ~0.8% exonic, ~45% intronic, ~41% intergenic, ~9% Up/Downstream and other small variants were detected (Supplementary Table 1).

The sequencing depth, base quality scores, GC content, duplication rates, base sequence content etc., are efficient and accurate QC filtering parameters for raw read sequence data. Unlike these QC parameters, the transition/ transversion (Ti/Tv), and heterozygous/nonreference-homozygous (het/hom), ratios cannot be used directly to filter individual SNPs but can rather be used to measure the overall SNP quality for high-throughput sequence data^43^.

**Fig. 5:**
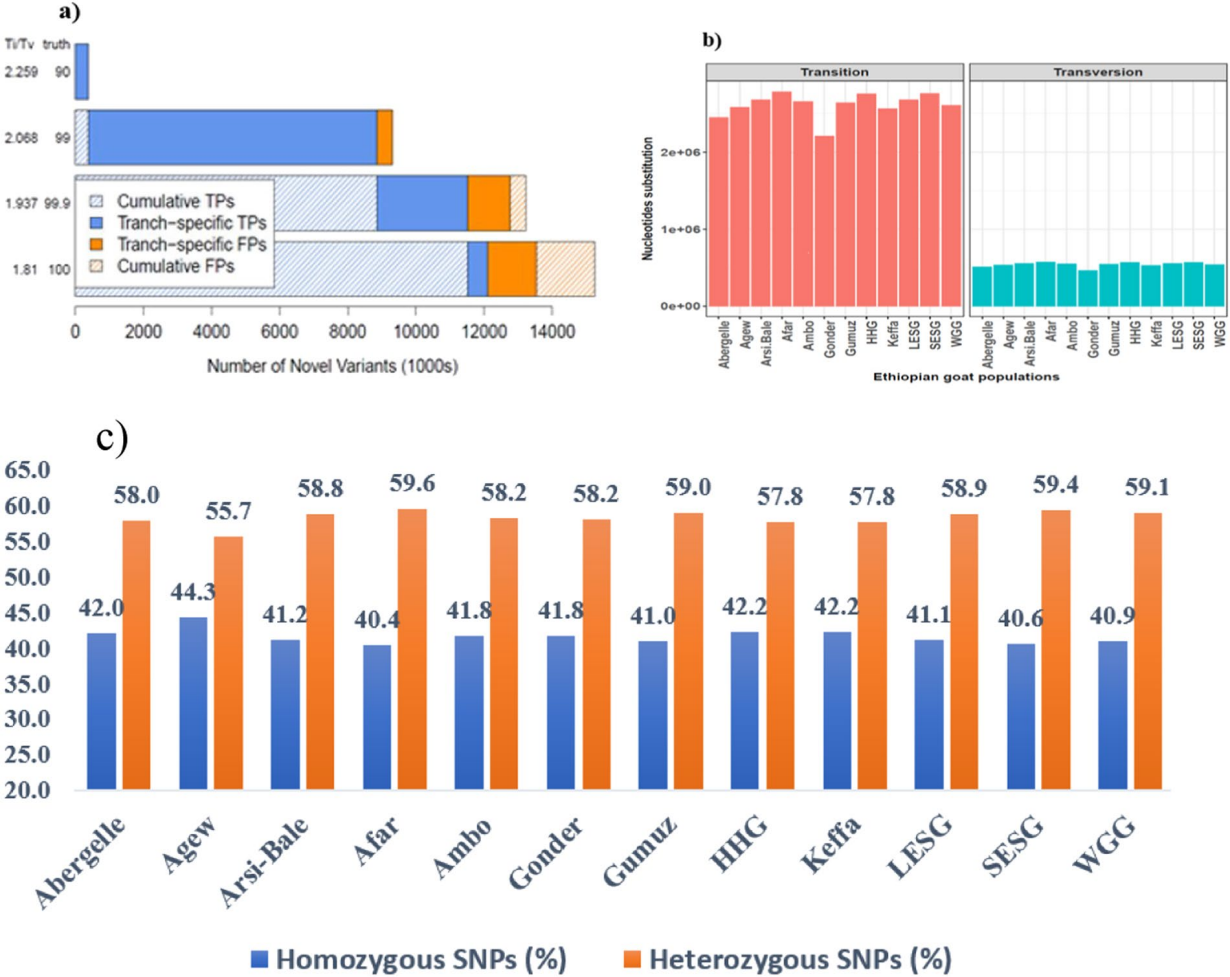
Quality control parameters using SNP data. (**a**) Tranches plot generated by VariantRecalibrator (VQSR). In this plot, the x-axis indicates the number of putative novel variants called true- and false-positive variants. In contrast, the y-axis shows two quality metrics: novel transition to transversion (Ti/Tv) ratio and the overall truth sensitivity, TPs= True-positives (the called variants in our callset and also present in the truth dataset), and FPs=False-positives (the called variants in our callset but not present in the truth dataset), (**b**) Nucleotide base substitution taking place in each goat population, and (**c**) Heterozygous/non-reference-homozygous (het/hom) ratio in each goat population.

In actual sequencing data, the Ti and Tv ratio is frequently above 0.5^43^. Inter-species comparisons^44^ and previous sequencing projects agree on a Ti/Tv ratio of ∼2.0–2.1 for genome-wide datasets^45^ while the expected values for this ratio for known and new variants are 2.10 and 2.07, respectively but a value of up to 2.4^44^ but not exceeding 4.0^38,43^ is acceptable. A significant deviation from the expected values could indicate artefactual variants resulting in biased estimates. Following VQSR filtration with the default tranche sensitivity threshold values (100.0, 99.9, 99.0 and 90.0%), the Ti/Tv ratio for our sequences ranged between 1.8 and 2.26 before the final filtration (Fig. 5a). Further filtration using ApplyRecalibration, with the tranche sensitivity threshold of 99.0% and restricting the alleles into biallelic SNPs, raised the ratio to 2.39 for the final SNP dataset. However, the Ti/Tv ratio varies with the genomic region (e.g. intronic, intergenic, exonic) but is not or is little affected by population ancestry^43^. Additionally, in each Ethiopian goat population, the transition mutation is more than twice the transversion mutations (Fig. 5b). However, the effects of the former on amino acid substitution are less detrimental than the latter^46^.

Similarly, under Hardy-Weinberg equilibrium assumptions, the expected value for the het/hom ratio in human WGS is estimated to be 2.0^40^. Population ancestry can affect the het/hom ratio but has not been observed to vary across the genome^43^. In our study, the het/hom ratio ranged from 1.26 in Agew to 1.48 in Afar goats (Fig. 5c). These ratios do not deviate much from that reported in humans (2.0) and is thus a good indicator of the quality of the sequences.

The SNP density is another important parameter for assessing sequence quality (Supplementary Table 2). A high SNP frequency, for example, two SNPs within 10 bp genomic distance, or within a short region of the genome, could indicate false-positive calls, possibly due to indels^40^. In our analysis, the SNP density and variant distribution for each chromosome were determined using VCFtools (v0.1.15) with the command line “–SNPdensity1000.” This command counted the number of variants found in each chromosome within a 1000 bp window size and the mean and standard deviation of the SNP density was computed using R software (v4.1.0)^47^. The tidyverse package in R was used to group and visualize the SNP density for each chromosome. The highest (11.42 ± 6.6 per kb) and lowest (8.66 ± 6.2 per kb) SNP density (mean one SNP in 0.01 kb) was observed in chromosome 28 and 18, respectively, which confirms the high-quality of our sequences.

### Supplementary information

Supplementary Table 1 and Table 2

## Data Availability

The steps from quality control to variant calling and refinement are presented below. *1*. ***FASTQC (v0.11.5): code for quality control for high throughput sequence data*** fastqc -t 8 /my_sample_R1. fastq.gz fastqc -t 8 /my_sample_R2. fastq.gz *2*. ***MulitQC (v1.8): Consolidate all the samples using “multiqc.”*** *3*. ***BWA-mem (0.7.17); code for mapping raw reads*** RGID = “ID_my_sample”, RGSM = “ID” bwa mem -t 8 -k 32 -M -R @RG\\tID: ${RGID}\\tLB:${RGSM}\\tPL:ILLUMINA\\tSM:${RGSM}${REF} ${input}/${RGID}.R1.fastq.gz ${input}/${RGID}.R2.fastq.gz | samtools view -bS - > ${my_sample}.bam *4*. ***Samtools (v1.8): code for sorting and indexing bam files*** samtools sort ${my_sample}. bam > ${my_sample}.sorted.bam samtools index ${my_sample}. sorted.bam -@ 8 *5*. ***Picard (v2.18.2)***: ***code for marking duplicate reads***: java -Xmx8G -jar ${picard}/picard.jar MarkDuplicates I = ${my_sample}.sorted.bam o = ${my_sample}_dedup.bam M = ${my_sample}_dedup.metrics.txt TMP_DIR = ${KNOWNVAR}/tmp MAX_FILE_HANDLES_FOR_READ_ENDS_MAP = 4000 OPTICAL_DUPLICATE_PIXEL_DISTANCE = 2500 CREATE_INDEX = true VALIDATION_STRINGENCY = LENIENT ***# To calculate the total number of clean reads, mapped and unmapped reads*** samtools flagstat ${my_sample}_dedup.bam > ${my_sample}_dedup.flagstat.txt *6*. ***GATK (v3.8-1-0-gf15c1c3ef): codes for Base Quality Score Recalibration (BQSR) steps*** ***# BQSR applies machine learning and builds a mode of covariation (true variation and artifacts) based on the input data and set of known variants as training resources and truth sets***. java -Xmx80G -jar ${GATK} -T BaseRecalibrator -R ${REF} -I ${my-sample}_dedup.bam -knownSites ${KNOWNVAR} -o ${my_sample}_recal_table ***#Apply the recalibration to your sequence data*** java -Xmx80G -jar ${GATK} -T PrintReads -R ${REF} -I ${my_sample}_dedup.bam -BQSR ${my_sample}_drecal_table -o ${my_sample}_recal.bam *7*. *GATK (v3.8-1-0-gf15c1c3ef):Codes for variant calling in GVCF mode by HaplotypeCaller* java -Xmx80G -jar ${GATK} -T HaplotypeCaller -R ${REF} -I ${my_sample}_recal.bam --genotyping_mode DISCOVERY --emitRefConfidence GVCF --variant_index_type LINEAR --variant_index_parameter 128000 -stand_call_conf 30 -o ${my_sample}_g.vcf.gz *8*. *GATK (v3.8-1-0-gf15c1c3ef): Joint genotyping for all individual VCF samples* ***# Use either --variant or -V options*** java -d64 -Xmx48g -jar ${GenomeAnalysisTK.jar} -T GenotypeGVCFs -R ${REF} --variant my_sample_g.vcf.gz --variant my_sample1_g.vcf.gz --variant my_sample2_g.vcf.gz --dbsnp ${KNOWNVAR} -o allsample_joint.vcf.gz *9*. ***GATK (v3.8-1-0-gf15c1c3ef): Code for VQSR steps*** java -d64 -Xmx48g -jar ${GenomeAnalysisTK.jar} -T VariantRecalibrator -R ${REF} -input ${allsample_joint}. vcf.gz -resource: dbSNP, known = false, training = true, truth = true, prior = 15.0${TRUEVAR} -resource: dbSNP, known = true, training = false, truth = false, prior = 2.0${KNOWNVAR} -an DP -an QD -an MQRankSum -an ReadPosRankSum -an FS -an SOR -mode SNP -tranche 100.0 -tranche 99.9 -tranche 99.0 -tranche 90.0 -recalFile ${allsample_joint)_recalibrate_SNP.recal -tranchesFile ${allsample_joint}_recalibrate_SNP.tranches -rscriptFile ${allsample_joint}_recalibrate_SNP_plots.R ***#Apply the SNP recalibration model to the variant call sets using ApplyRecalibration GATK walker***. java -d64 -Xmx48g -Djava.io.tmpdir = **$**{allsample_joint.vcf}/javatempdir -jar ${GenomeAnalysisTK.jar} -T ApplyRecalibration -R ${REF} -input ${allsample_joint). vcf.gz --ts_filter_level 99.0 -mode SNP -tranchesFile ${allsample_joint}_recalibrate_SNP.tranches -recalFile ${allsample_joint}_recalibrate_SNP.recal -o ${allsample_joint}_snp_VQSR_ApplyRecal_filtered.vcf.gz ***#Post-processing to remove variants failing the GATK filtering parameters and restricting the alleles into biallelic markers only*** *.* java -d64 -Xmx48g -jar ${GenomeAnalysisTK.jar} -R ${REF} -T SelectVariants --variant ${allsample_joint} _snp_VQSR_ApplyRecal_filtered.vcf.gz -o ${final_filtered}. vcf.gz -selectType SNP -env -ef -restrictAllelesTo BIALLELIC
