# Supplementary material for: Whole-genome resource sequences of 57 indigenous Ethiopian goats
Source: Sci Data. 2024 Jan 29;11:139. doi: 10.1038/s41597-024-02973-2 (PMC10825132; doi:10.1038/s41597-024-02973-2)
Supplement: Supplementary file 1 — Supplementary Table 1 and Table 2 [file 41597_2024_2973_MOESM1_ESM.pdf]

Supplementary Table 1. Summary of the whole-genome variants found in the Ethiopian indigenous goat populations

**Category**	**Abergelle**	**Agew**	**Arsi-Bale**	**Afar**	**Ambo**	**Gonder**	**Gumuz**	**HHG**	**Keffa**	**LESG**	**SESG**	**WGG**
Total SNPs	13,000,136	13,632,756	14,091,056	14,619,159	13,980,014	11,804,167	13,905,168	14,493,364	13,543,509	14,117,284	14,506,560	13,747,534
Novel	2,864,191	3,026,005	3,143,629	3,283,048	3,115,559	2,560,390	3,091,963	3,243,918	2,999,254	3,154,629	3,246,357	3,058,703
Deletions	644,040	1,214,937	698,674	718,025	691,245	585,895	691,533	712,020	672282	698,004	714,295	681,593
Insertions	785,384	1,727,263	867,965	899,265	859,347	698,951	857,645	890,758	828,994	868,119	893,093	843,123
**Annotated Variants**												
Splicing	573	578	583	579	575	551	587	615	580	580	573	594
Missense	51,623	54,854	56,477	58,063	56,206	46,244	56,254	57,721	54,073	56,090	57,856	55,009
Synonymous	88,390	93,204	95,614	99,011	95,390	80,224	94,809	97,976	91,804	94,978	98,425	92,777
Stop gained/ lost	618	679	666	669	668	660	675	664	661	662	669	672
Coding	29	34	31	28	39	34	37	35	32	40	34	43
Intronic	8,683,340	9,101,591	9,387,391	9,740,194	9,319,594	7,857,220	9,283,497	9,643,993	9,014,120	9,380,759	9,681,510	9,154,378
ncRNA exonic	26,942	28,492	29,550	30,734	29,628	24,378	28,819	30,307	27,902	29,406	30,411	28,656
ncRNA intronic	811,028	851,438	884,271	909,356	878,978	736,857	870,603	908,202	840,082	880,031	904,719	858,028
ncRNA splicing	16,552	17,472	18,114	18,718	17,899	14,851	17,869	18,677	17,287	18,076	18,728	17,594
UTR3/UTR5	77,188	81,046	82,197	84,804	81,727	70,588	81,552	84,089	79,804	82,500	84,134	80,191
Up/downstream	1,696,923	1,741,340	1,771,705	1,803,865	1,764,098	1,619,714	1,759,813	1,799,413	1,735,994	1,768,786	1,798,567	1,744,769
Intergenic	7,826,146	8,205,750	8,484,285	8,802,491	8,416,255	7,115,115	8,364,134	8,723,883	8,156,620	8,508,103	8,730,533	8,293,664

Supplementary Table 2: Autosomal biallelic SNPs in each chromosome and its SNP density

Chromosome	SNP counts	SNP density (count/Kb)
1	1,641,907	10.431±6.04
2	1,341,460	9.827±5.76
3	1,141,937	9.513±5.73
4	1,208,748	10.012±5.92
5	1,145,033	9.621±5.87
6	1,282,676	10.903±6.30
7	1,038,416	9.577±6.16
8	1,105,377	9.811±5.74
9	906,140	9.897±5.73
10	936,877	9.270±5.82
11	998,815	9.404±5.49
12	962,655	11.030±7.40
13	760,763	9.162±5.39
14	981,783	10.370±6.26
15	871,598	10.642±6.58
16	802,126	10.107±6.13
17	718,003	10.094±5.99
18	582,525	8.659±6.17
19	580,399	9.284±6.20
20	787,808	10.975±5.57
21	697,902	10.053±6.35
22	561,653	9.317±5.57
23	554,990	11.358±8.81
24	653,102	10.483±5.89
25	411,698	9.606±5.71
26	543,158	10.564±6.32
27	477,246	10.675±6.30
28	509,977	11.418±6.60
29	554,807	10.808±6.29
